# Fluoroscopy Assisted Minimally Invasive Transplantation of Allogenic Mesenchymal Stromal Cells Embedded in HyStem Reduces the Progression of Nucleus Pulposus Degeneration in the Damaged Interverbal Disc: A Preliminary Study in Rabbits

**DOI:** 10.1155/2014/818502

**Published:** 2014-03-30

**Authors:** Rifa Aquidah Subhan, Karunanithi Puvanan, Malliga Raman Murali, Hanumantha Rao Balaji Raghavendran, Samuel Shani, Basri Johan Jeet Abdullah, Azlina Amir Abbas, Jamal Azmi Mohamed, Tunku Kamarul

**Affiliations:** ^1^Tissue Engineering Group, NOCERAL, Department of Orthopaedic Surgery, Faculty of Medicine, University of Malaya, 50603 Kuala Lumpur, Malaysia; ^2^Department of Biomedical Imaging, Faculty of Medicine Building, University of Malaya, 50603 Kuala Lumpur, Malaysia

## Abstract

This study was conducted to develop a technique for minimally invasive and accurate delivery of stem cells to augment nucleus pulposus (NP) in damaged intervertebral discs (IVD). IVD damage was created in noncontiguous discs at L4-L5 level; rabbits (*N* = 12) were randomly divided into three groups: group I treated with MSCs in HyStem hydrogel, group II treated with HyStem alone, and group III received no intervention. MSCs and hydrogel were administered to the damaged disc under guidance of fluoroscopy. Augmentation of NP was assessed through histological and MRI T2 mapping of the NP after eight weeks of transplantation. T2 weighted signal intensity was higher in group I than in groups II and III (*P* < 0.05). Disc height index showed maximum disc height in group I compared to groups II and III. Histological score of the degenerative index was significantly (*P* < 0.05) lower in group I (8.6 ± 1.8) than that in groups II (11.6 ± 2.3) and III (18.0 ± 5.7). Immunohistochemistry staining for collagen type II and aggrecan staining were higher in group I as compared to other groups. Our results demonstrate that the minimally invasive administration of MSCs in hyaluronan hydrogel (HyStem) augments the repair of NP in damaged IVD.

## 1. Introduction

Intervertebral disc (IVD) degeneration is a complex process that produces an accelerated unfavorable, adverse effect on IVD morphology, histology, and function in many patients with chronic back pain [1, 2]. It continues to be a major cause of low back pain, incapacitating the function of any individual, which when left uncorrected not only leads to significant morbidity but also results in substantial economic burden [3]. In recent years the trend of treating IVD has shifted from treating it symptomatically using pharmaceutical agents, various instrumentations, or fusion to a more curative alternative such as disc replacements. These were done with the aim of rejuvenating the damaged disc tissue and providing long-term sustainable outcome for patients [4]. Unfortunately, many studies have demonstrated that the use of such treatments resulted in many complications in addition to limiting the life span [5]. Hence, the current research has been focused on finding better alternatives among which the use of biological methods to repair degenerated IVDs like gene therapy, growth factor injections, cell-based tissue engineering, and cell therapy is gaining much importance [6–8].

Several* in vivo* studies have used notochordal nucleus pulposus (NP) cells, autologous disc chondrocytes, and mesenchymal stromal cells (MSC) to generate new disc formation. However, these studies demonstrated variable results [9, 10]. Study by Jandial et al. showed promising results suggesting a possibility to slow down the process of degeneration and even result in the regeneration of NP employing such techniques [11]. However, further investigations on animal models are needed to validate these findings. These will help to define the effective time window for the treatment of progressive IVD degeneration and to identify appropriate donor cells and scaffold material for use in such conditions [12].

IVDs are specialized structures that link adjacent vertebral bodies and contribute to the flexibility of the spine whilst functioning as “shock absorbers” for the spine. Each IVD is comprised of a central, gelatinous proteoglycan rich nucleus pulposus (NP), constrained by a fibrous annulus fibrosus (AF) and joined to the adjacent vertebrae by cartilaginous end plates. In IVD degeneration, distinct changes like progressive loss of proteoglycans and water content in the NP, filling of the NP space with fibrocartilage, delamination of the AF, and osteophyte formation in the adjacent vertebral end plates can be observed [2].

In achieving a potent cure for disc degeneration, restoration of the normal cellular constituents of the tissue is crucial. As the main pathology lies within the NP, biological therapies that provide a means of producing normal NP cells with a suitable environment are usually preferred. It has been shown that the differentiation of MSCs to a diverse set of cells, including NP, AF, articular cartilage, and auricular cartilage, depends primarily on the environment in which they are placed [9, 10]. Hence it is difficult to direct the differentiation of cells into a certain lineage, in particularly NP cells, when the environment within the IVD itself is mixed. It has been suggested that the use of certain matrices such as those mimicking the nucleus pulposus (NP) may provide the much needed environmental milieu for NP cells to thrive and function as would those in normal IVD. In several studies, the use of collagen mixed substances that form a gel like material that has the consistency of NP has been shown to maintain the phenotype of NP cells [13]. Furthermore, other studies using similar matrices in damaged discs in animal models appear to produce desirable reparative effects [14]. It is thus fair to hypothesize that a combination of NP or progenitor cells embedded in such materials implanted in damaged IVD may repair or even replace these damaged tissues. However, it has been reported that transplanted cells can leak from the intervertebral space, which results in the formation of undesirable bone spurs (osteophytes), severely complicating the restorative processes [15]. In this context, precise delivery of stem cells in hydrogel into the damaged disc is of much importance. The present study was therefore conducted to prove our hypothesis by assessing the effectiveness of minimally invasive delivery of MSCs embedded in the hyaluronan based hydrogel (HyStem) in augmenting damaged nucleus pulposus.

## 2. Materials and Methods

### 2.1. Study Design

Twelve (*N* = 12) New Zealand white rabbits, 3-4 months old, weighing 2.6–3.2 kg, were used in this study. The manner in which the study was conducted strictly followed the guidelines of the Animal Care and Use Committee and Institutional Review Board, University of Malaya (reference number: OS/13/12/2010/PK(R)). Disc damage was created in all the animals through annular puncture at level L4-L5. The animals were randomly divided into three groups with four animals each. The groups were as follows: group I—MSCs transplanted with HyStem (Sigma-Aldrich–St. Louis, USA), group II—transplantation of HyStem alone, and Group III—damage left untreated.

### 2.2. Creation of Intervertebral Disc Damage

**Figure 1:**
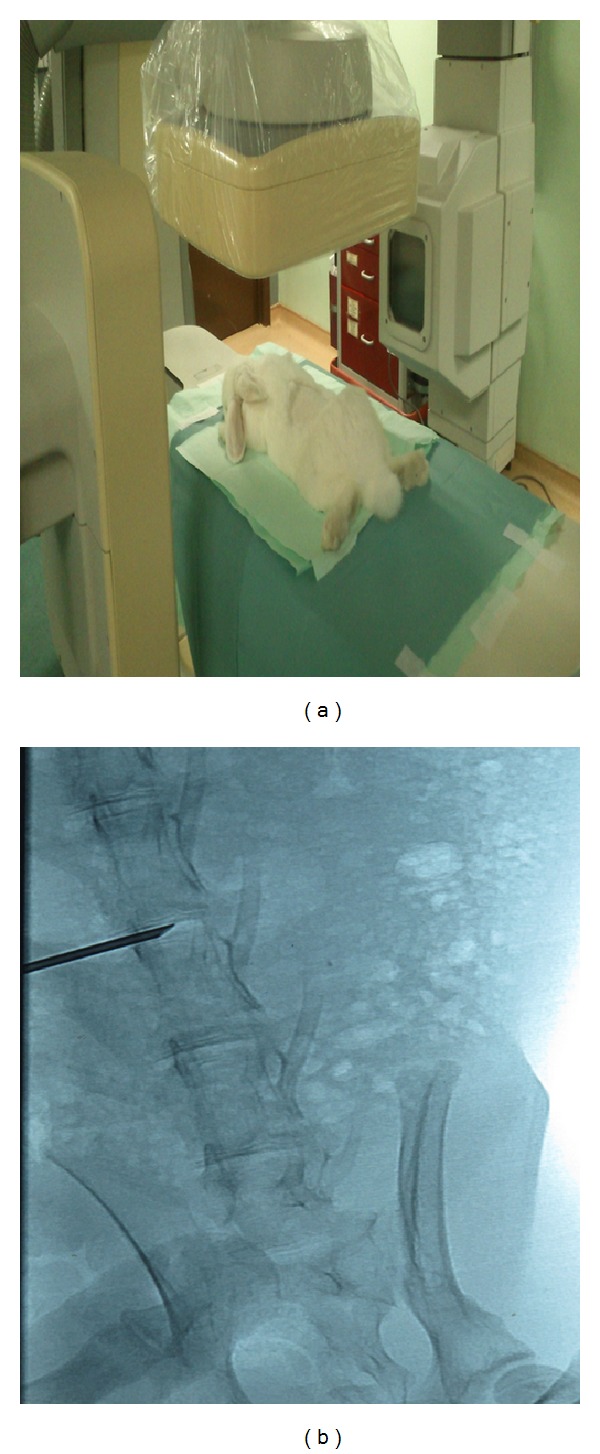
Rabbits placed under the fluoroscopy machine (a). Image captured after the disc damage was created at IVD L4/L5 (b).

Single level damage of the L4/L5 IVD was induced through fluoroscopy-guided percutaneous needle puncture technique [16]. Single disc level was chosen to avoid other factors that can alter the findings such as adjacent segment syndrome. Rabbits were anaesthetized by an intramuscular injection of ketamine hydrochloride (25 mg/kg) mixed with xylazine (5 mg/kg) and the site was shaved and cleaned. Under fluoroscopic guidance (Philips), the rabbits were placed in prone position and the spine was approached posterolaterally (Figure 1(a)). The L4/L5 IVD was identified by counting the IVDs proximal to distally starting from the lowest thoracic vertebrae. An 18-gauge needle was aseptically introduced through the left posterolateral approach 2.5 cm from the midline spinous process at a 30°–45° angle from midline to lateral (Figure 1(b)). This was carefully advanced ensuring no damage to end plate, until it reached the center of the IVD. After confirming the position, NP was scratched by rocking the needle five times.

Postoperatively, the rabbits were then monitored till they awoke and were then allowed to roam freely in their cages. They were given intramuscular Kombitrim and IM meloxicam for postoperative analgesia every 24 hours for 3 days.

### 2.3. MSC Isolation and Culture

Allogenic MSCs were obtained from the femur and tibia of four rabbits. Briefly, 2 mL of bone marrow was diluted with 2 mL PBS solution and layered over 3 mL Ficoll-paque (GE Healthcare, Amersham Biosciences, Piscataway, NJ, USA) in a 15 mL Falcon tube (Corning, NY, USA). Mononuclear cells were harvested from the interface of plasma and Ficoll-paque after centrifugation at 2,200 rpm for 25 min. The cells were then washed with prewarmed DMEM (Invitrogen, Carlsbad, USA) and centrifuged for another 10 minutes. The cell pellet was resuspended in DMEM containing 10% FBS (Invitrogen, Carlsbad, USA) and 1% penicillin-streptomycin (Invitrogen, Carlsbad, USA), seeded into a T75 flask, and incubated at 37°C in a humidified environment of 5% CO2. After 8 days, the nonadherent cells were removed, while the adherent cells (alloMSCs) were replenished with fresh medium. The allogenic MSC culture was maintained in monolayer for 3 weeks and transplanted at passage 2 [17].

### 2.4. Characterization of MSC

To ensure that the isolated cells consisted of defined homogeneous population of MSC, expression of surface markers in MSC cultures at passage 3 was analyzed using immunohistochemical staining. Cells were seeded at a density of 10,000 cells per chamber in 4-well chamber slides and the protocol followed was according to the manufacturer's instructions (Dako, Denmark). Briefly, MSCs were fixed in 4% paraformaldehyde/PBS for 15–20 min and then blocked for 30 min using hydrogen peroxide (H_2_O_2_) to prevent endogenous peroxidase activity. MSCs were then incubated in goat serum working solution for 15 min to block non-specific binding. MSCs were incubated with primary antibody CD29 mouse anti-rabbit monoclonal antibody (Chemicon, Billerica, USA), CD 44 mouse anti-rabbit monoclonal antibody (Genetex, Alton Pkwy, Irvine, USA), and CD45 mouse anti-rabbit monoclonal antibody (Abcam, Cambridge, UK) with 1 : 100 dilution at room temperature for 30 min. After washing with PBS, cells were incubated with secondary antibody at 1 : 200 dilution for 30 min. Cells were then washed with PBS, stained with DAB chromogen substrate, and examined under light microscopy (Nikon Eclipse TE2000-S, Nikon Corporation, Tokyo, Japan).

### 2.5. *In Vitro* Lineage Differentiation

The multipotent capacity of rabbit MSCs was proven after* in vitro* culturing with specific supplements by inducing differentiation into osteogenic, chondrogenic, and adipogenic. To induce osteogenic differentiation, confluent passaged-2 cells were cultured in the osteogenic medium (Invitrogen, Gibco, USA). After 21 days, Alizarin Red staining was used to observe the matrix mineralization. STEMPRO Chondrogenesis Differentiation Kit (Gibco, Invitrogen) was used to induce chondrogenic differentiation of rabbit MSCs in a micromass pellet culture system. Feeding of chondrogenic medium was carried out over a period of 21 days and the pellet was then processed for histology staining using Safranin O/fast green solution. For adipogenesis, adipogenic medium (Invitrogen, Gibco, USA) was used to induce the differentiation in the confluent culture of passaged-2 cells. Fourteen days after culture initiation, the cells were fixed with methanol at room temperature for 10 minutes, rinsed by 60% isopropanol, and stained by using freshly prepared Oil Red O solution in 99% isopropanol for 15 minutes. The images at different magnifications were captured using a camera attached to the light microscope (Nikon Eclipse E200, Nikon, Japan).

### 2.6. Preparation of MSC-HyStem Hydrogel and Transplantation

Since hyaluronic acid is one of the main components of IVD, HyStem hydrogel made of a synthetic hyaluronic acid-based matrix was used as a carrier. HyStem offers complete control of the cellular environment. Allogenic MSCs at a density of 1 × 10^6^ cells/mL embedded in HyStem hydrogel were transplanted after 8 weeks to the degeneration-induced L4-L5 discs in anesthetized rabbits. HyStem hydrogel gel-medium solution (0.04 mL), in which allogenic MSCs were embedded, was injected through 27-gauge Hamilton syringe to the damaged disc. The needle was then withdrawn slowly to prevent disc tear or leakage.

### 2.7. Scanning Electron Microscopy (SEM) Examination

The cell-hydrogel complexes were washed with PBS, fixed in 2.5% (v/v) glutaraldehyde in 0.1 M sodium cocodylate buffer containing 2% tannic acid, and kept at 4°C followed by postfixation using 2% osmium tetroxide for 2 h. The samples were then dehydrated through a graded ethanol series (35, 50, 70, 80, 90, and 95%) for 15 min each and twice at 100%. This process then continued by immersing in pure acetone for 15 min each. After dehydration, the samples were critical point dried (BAL-TEC, Uhingen, Germany) and gold coated ready to undergo scanning electron microscope examination (JSM-6400, JEOL, Japan).

### 2.8. Imaging Analysis

The rabbits were anesthetized by intramuscular injection of xylazine (Bayer, Puteaux, France) and ketamine (Merial, Lyon, France). Imaging analysis was done using a 3T imager (Signa MRI, General Electric Medical Systems, Florence, SC, USA) with the rabbits in supine position and the lumbar region centered over a quadrature coil normally used for human cervical spine. T2 weighted sagittal images were studied by a radiologist by measuring the intensity emitted in the intervertebral disc region. The T2 weighted midsagittal images of the intact and stabbed discs were analyzed qualitatively for evidence of degeneration. The Dicom-formatted image data (Dicom 3.0) was transferred to the Picture Archiving and Communications System (PACS) which was furnished with a Agfa IMPAX 3.5 Software. Region of Interest (ROI) measurements of the NP were obtained and quantitative analysis of these images was done using the PACS. The area and average signal intensity of this ROI were then computed automatically by IMPAX software and data downloaded to computer spreadsheet for analysis. Change in the disc high index of injected discs was expressed as percentage DHI (%DHI) and normalized to the measured preoperative IVD height: % DHI = (postoperative DHI/preoperative DHI) × 100. The MRI data of the disc, 8 weeks before transplantation and 16 weeks after transplantation, were normalized to the disc's corresponding preoperative data. Mean and standard deviation of the signal intensity from pre- and postoperative group was calculated.

### 2.9. Histology and Immunohistochemistry

The specimens were fixed in 10% buffered formalin overnight and then decalcified using 10% formic acid. The specimens were subsequently dehydrated in ethanol in a stepwise manner from 70% up to 100%, transferred to xylene, and embedded in paraffin. 4 *μ*m paraffin sections were prepared longitudinally. The samples were stained with hematoxylin and eosin to evaluate the cellular architecture, and Safranin O/fast green was used to detect proteoglycan. To detect the collagen and aggrecan distribution in the repair tissue, the specimen section slides were incubated in primary antibody solution (mouse anti-rabbit collagen type II, Merck, USA) (mouse anti-rabbit aggrecan, Abcam, England), followed by secondary antibody goat anti-mouse with FITC labelled. Histological specimens were scored following the scale specified in the modified Boo scoring system [18]. Histological analysis of the repair tissue was performed by three individual observers blinded to the sample origin.

### 2.10. Statistical Analysis

The overall differences for each parameter were determined using nonparametric analyses, that is, Kruskal-Wallis and Mann Whitney *U* tests, to evaluate the level of significance between the groups. *P* values of less than 0.05 were considered significant. Statistical analyses were performed using SPSS version 17 (SPSS Inc., Chicago, IL, USA).

## 3. Results

### 3.1. Characterization and Differentiation

**Figure 2:**
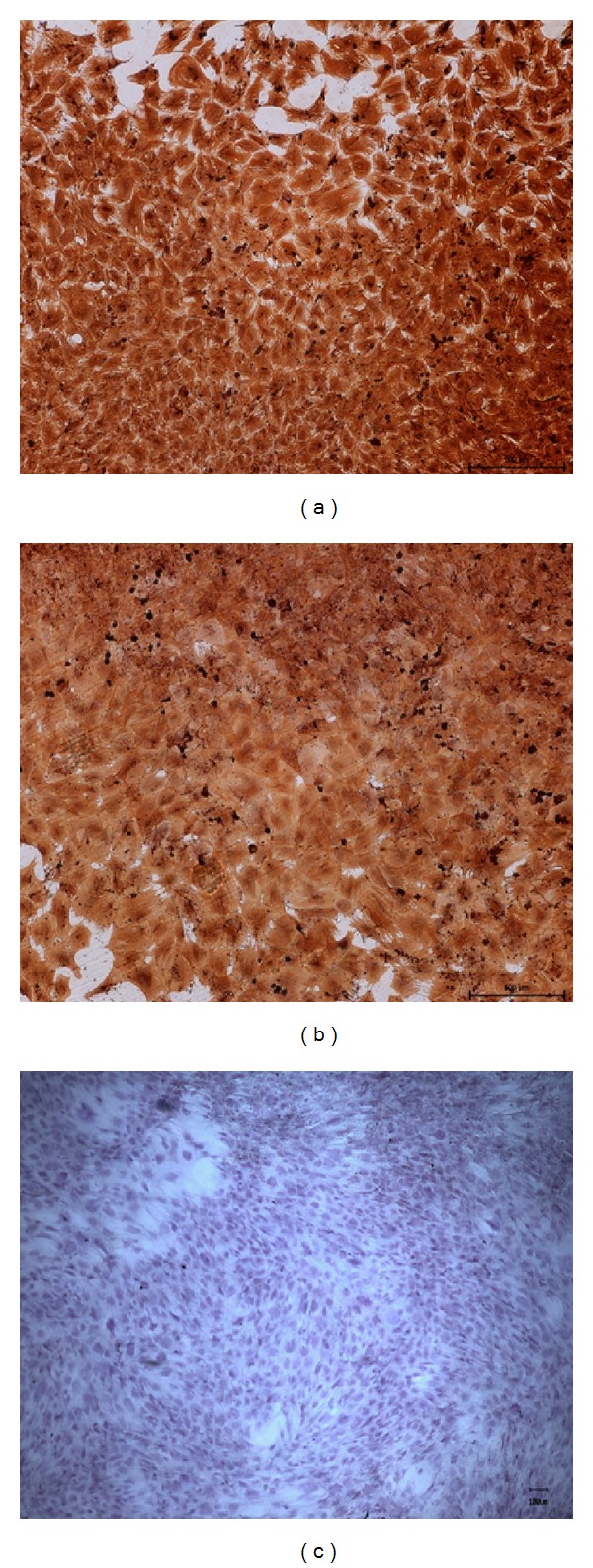
Immunohistochemical staining of MSCs. CD 44 positive marker (a), CD 29 positive marker (b), and CD 45 negative marker (c).

The cells showed abundant CD44 (Figure 2(a)) and moderate CD29 (Figure 2(b)) expressions, both of which are the generally accepted markers for MSCs. In contrast, no expression of the hematopoietic lineage markers CD45 (Figure 2(c)) was observed in the cultures.

### 3.2. *In Vitro* Lineage Differentiation

**Figure 3:**
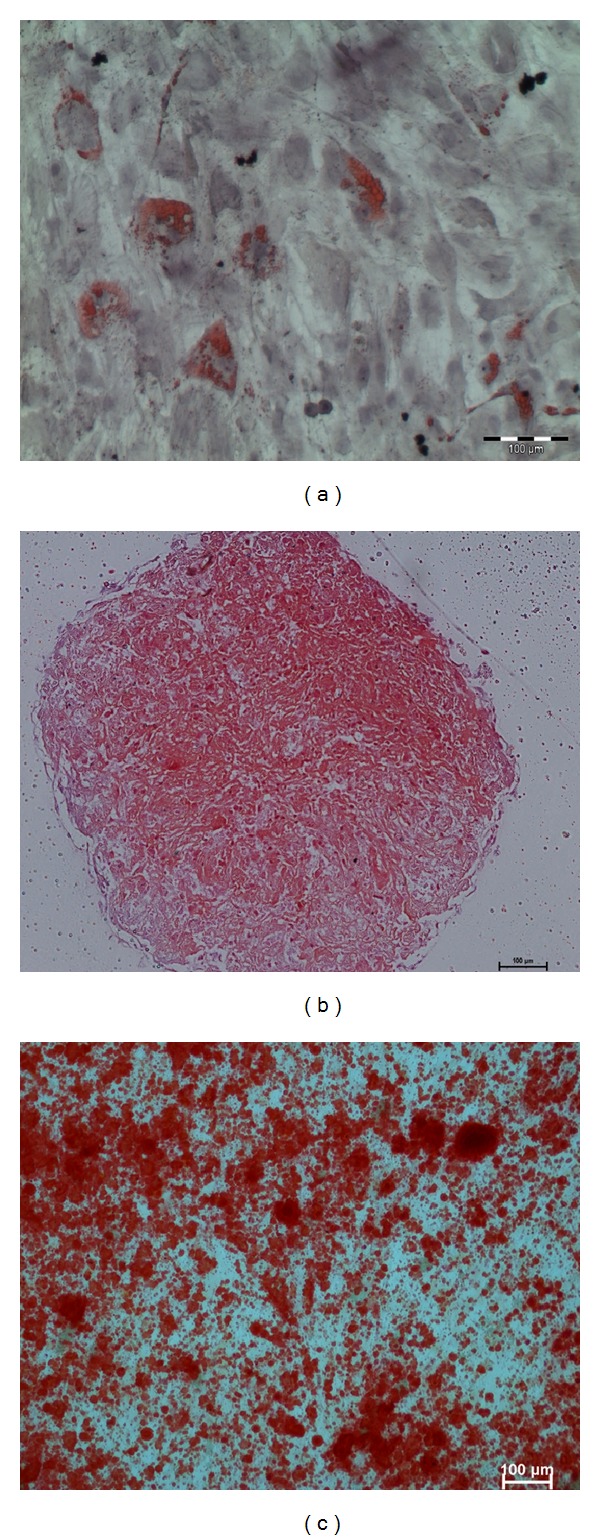
The functional assays for differentiation of culture MSCs. (a) Representative images of adipogenic differentiation demonstrated by formation of oil droplet. (b) Chondrogenic differentiation demonstrated by positive expression of proteoglycans matrix within the pellet stained with Safranin O/fast green staining. (c) Osteogenic differentiation demonstrated by positive bone matrix mineralization stained with Alizarin Red S staining.

In adipogenic culture, small lipid droplets appeared within the cytoplasm of the cells by day 3 and day 4. They gradually occupied the whole cell by day 14. On staining with Oil Red O, the lipid droplets stained red (Figure 3(a)). In the micromass culture system for chondrocyte differentiation, the size of the pellet seemed to be increased during the culture period, probably as a result of matrix production and secretion. Metachromatic nature of the matrix was demonstrated by Safranin O staining (Figure 3(b)). In osteoinductive cultures, nodule-like structures were observed in certain regions. On staining with Alizarin Red, the regions of mineralization were distinctly stained red, which indicate the early stage of bone formation (Figure 3(c)). These observations suggested that MSCs have the potential to differentiate between chondrogenic, osteogenic, and adipogenic lineages.

### 3.3. Scanning Electron Microscopy (SEM) Examination

**Figure 4:**
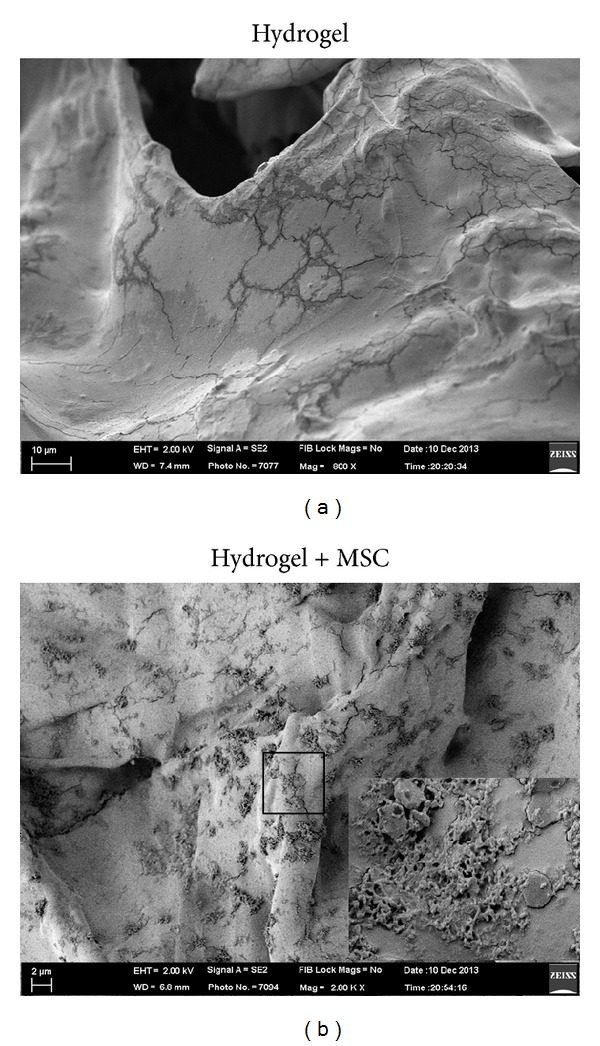
SEM image of Hyaluron based hydrogel alone (a). Cell attached on the surface of hydrogel (b).

Figure 4(a) shows structure of hydrogel form after crosslink. The hydrogel material was proven to support attachment of cells (Figure 4(b)). The cells appeared to be round as they were attached.

### 3.4. Imaging Analysis

**Figure 5:**
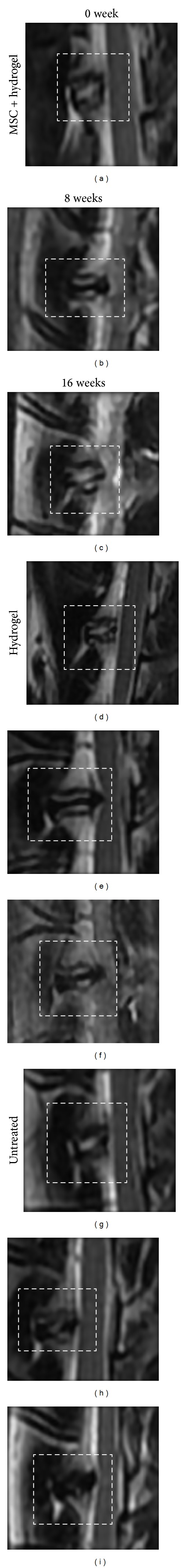
MRI images (T2 weighted sagittal views) before operation, 8 weeks, and 16 weeks after annular puncture was created. MSCs containing hydrogel group I baseline (a), 8 weeks (b) and 16 weeks (c), showing improvement in signal intensity suggesting regeneration of IVD. Hydrogel only group II baseline (d), 8 weeks (e) and 16 weeks (f), showing progression of disc degeneration. Untreated group III baseline (g), 8 weeks (h) and 16 weeks (i), showing progression of degeneration.

**Figure 6:**
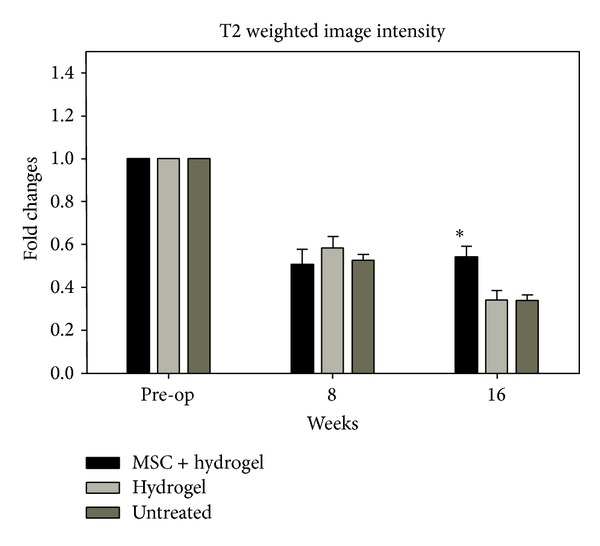
Magnetic resonance imaging results of normalized T2 values. Magnetic resonance imaging (MRI) analysis was performed before operation, 8 weeks after defects were created, and 8 weeks after transplantation (16 weeks). The normalized T2 value of group I after transplantation with MSC + hydrogel shows significant difference between group II and group III. ∗ indicates significance at *P* < 0.05.

**Figure 7:**
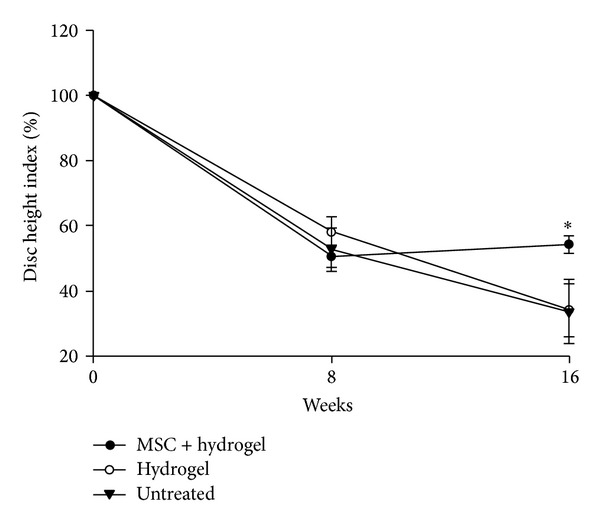
Nucleus pulposus disc height index. Values obtained at each point are normalized to preoperative score at day “0” (100%). ∗ indicates significance at *P* < 0.05.

Representative MRI image of control IVD in Figures 5(a), 5(d), and 5(g) indicated a normal T2 weighted intensity. Eight weeks after creation of defect (Figures 5(b), 5(e), and 5(h)) the T2 signal intensity index decreased in all of the 3 groups. However, after 16 weeks the index in group I (Figure 5(c)) of MSC containing hydrogel transplantation showed significant difference (Figure 6) (0.5 ± 0.04) (*P* < 0.05) compared to group II (Figure 5(f)) with hydrogel insertion (Figure 6) (0.3 ± 0.4) and group III (Figure 5(i)) which is untreated (Figure 6) (0.3 ± 0.02). In all groups, 8 weeks after the initial annular puncture showed a significant narrowing of disc height compared with that of the nonpunctured normal disc (Figure 7) (approximately 50.0% decrease compared with the baseline percentage DHI values before annular puncture, *P* < 0.05). There were no significant differences in the percentage DHI among the experimental groups. The treatment affected the postinjection disc. Eight weeks after transplantation with MSC containing hydrogel, group I disc height began to recover (54% ± 2.6%) compared with the disc injected with hydrogel group II (34% ± 8.0%) and untreated group III (33% ± 9.8%) (*P* < 0.05).

### 3.5. Histology and Immunohistochemistry

**Figure 8:**
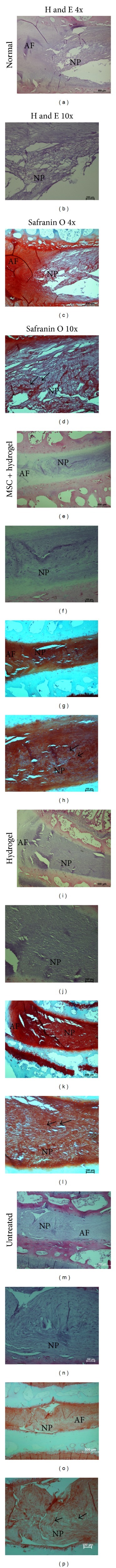
Typical histological changes in normal disc (a–d), MSC and hydrogel (e–h), hydrogel (i–l), and untreated (m–p). On Safranin O stained sections, a moderate-to-severe degeneration was observed in the hydrogel (k, l) and untreated (o, p) groups with formation of fibrocyte-like cells. In the MSC containing hydrogel group, the increasing number of chondrocyte-like cells which appeared as large cells encircled with pericellular matrix densely stained with Safranin O was found in the nucleus pulposus (g, h).

**Figure 9:**
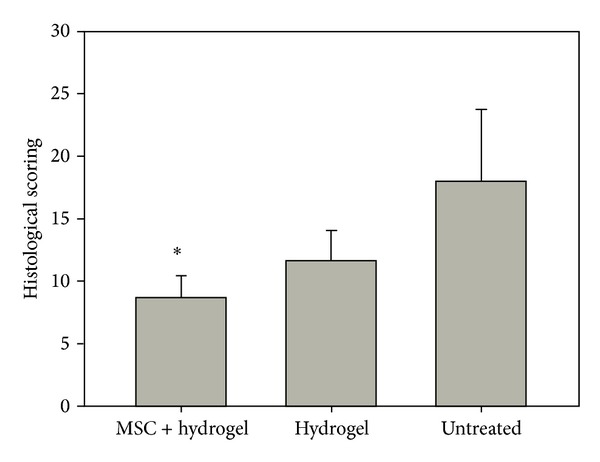
Quantitative histological evaluation of disc degeneration index using Boo's scoring. Significance is represented by *(*P* < 0.05).

**Figure 10:**
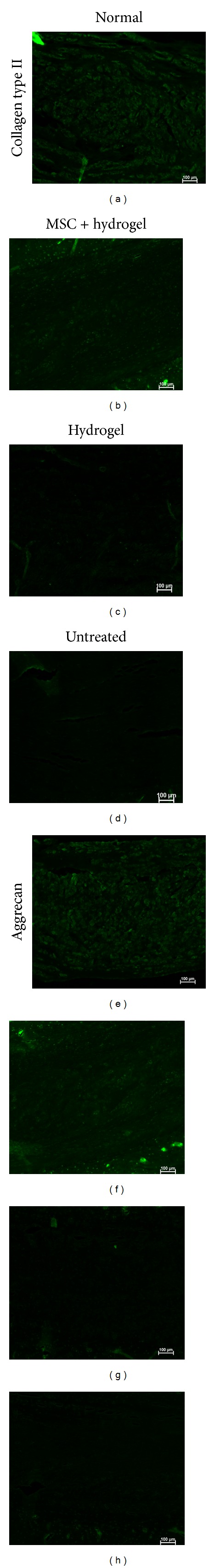
Immunohistochemical staining of different groups stained for collagen type II and aggrecan. Normal group (a, e), MSC and hydrogel group (b, f), hydrogel group (c, g), and untreated defect group (d, h).

In the normal disc (Figures 8(a) and 8(b)) stained by H and E, the boat-shaped NP was comprised of distinct cell type from AF. The AF was intact and the border between the AF and the NP was clearly defined. Safranin O stained histology in the normal group (Figures 8(c) and 8(d)) showed a homogenous distribution of stain. In the untreated group III, NP area decreased and became irregular wavy fibrocartilage lamella and was associated with fibrochondrocyte-like cells of the AF (Figures 8(m)–8(p)). Discs in the hydrogel-alone treated group II still displayed features of disc degeneration to a certain extent (Figures 8(i)–8(l)). Conversely, the cell distribution and the extracellular matrix alignment in the disc treated with MSC containing hydrogel group I (Figures 8(e)–8(h)) were more even and regular than those in the untreated and hydrogel-alone groups. The NP area in the intact disc stained strongly with Safranin O with the increasing numbers of chondrocyte-like cells, which appear as large cells encircled with pericellular matrix. However, the stained levels were not homogenous, as some regions stained very densely compared with surrounding vicinities. The Safranin stained area in the nontreated group was much smaller and weaker. In all of the experimental groups, the penetration of blood vessels or inflammatory cells was not observed within the discs. Histological score (Figure 9) of the degenerative index was significantly (*P* < 0.05) lower in the MSC-hydrogel treated group (8.6 ± 1.8) than that of the hydrogel alone (11.6 ± 2.3) and the untreated defect group (18 ± 5.7). The lower index of histological scoring in MSC-hydrogel group indicated improvement in disc repair. Immunohistochemical staining for collagen type II and aggrecan for MSC-Hydrogel treated group as shown in Figure 10 showed a relatively denser staining compared to the hydrogel-alone and untreated group.

## 4. Discussion

Treatment of damaged discs continues to be an area of ongoing research with the focus today increasingly concerted towards tissue regeneration through the use of cellular therapies. This is mainly due to its promise of recreating the cellular and microenvironment constituents seen in normal IVDs as opposed to merely symptomatic relieving procedures that is being offered by the present day treatment options. The initial aim for the use of such therapies is to halt the disease progression, but, subsequently, it is hoped that such therapy will result in reversing the process of disc degeneration [19]. The use of MSCs in the present study was appropriate since these cells have the ability to undergo multilineage differentiation and are also highly viable* in vitro*. Their uses in several modality of treatment have shown to yield positive results [20]. It is therefore understandable that many studies have used MSCs in treating damaged IVDs in animal models. In several studies, the reversal of such degenerative process has been shown to be promising [12, 21]. Due to its intricacy in forming young and healthy nucleus pulposus cells, mesenchymal stem cells, in particular, have been recognized as the most likely candidate that will result in NP repair when used* in vivo*. It has been shown that, introducing MSCs into the damaged and hypoxic environment of NP, MSCs differentiate into chondrocyte-like cells native to NP both* ex vivo* and* in vivo* [22–24]. Although it may appear to be a simple procedure, injecting cells into IVD has been shown to be difficult due to various reasons [25]. Since the IVD environment is relatively harsh there is the problem of low survival of injected MSC* in vivo,* thus resulting in poor tissue regeneration [25]. Another study has also reported that, when injected, MSCs in suspension tend to leak into the surrounding disc, which leads to the osteophyte formation [15]. Due to these issues, the use of a carrier or scaffold which is delivered in minimal invasive way may be prudent. In this study, we focused on establishing a technique for minimally invasive MSC injection into the nucleus pulposus, using accurate fluoroscopy visualization. Our method of cell delivery was based on the introduction of MSCs, suspended in hydrogel, into the NP region. This also diminishes the risk of cell leakage. Preventing leakage of implanted cells is especially important for the clinical translation of this method. The choice of hydrogel was due to the fact that it has been shown that not only does this material prevent MSCs from leaking, but it also provides a framework for MSC growth and differentiation. In addition, the hyaluronic acid present in the implanted hydrogel has properties that mimic NP; that is, it aids in the absorption of water. This feature is important since the flow of fluid during mechanical loading will influence the dynamics of cellular nutrition and hydrational status [26]. This appears to be achieved by the cell-matrix material suggested in our study, as injection of mesenchymal stromal cells embedded in Hyaluron based hydrogel resulted in lower degenerated disc compared to the untreated defects.

MRI serves as an important tool in diagnosing and grading the severity of IVD degeneration as well as in monitoring the response to the treatment. The pictures of rabbit lumbar spines showed progressive degeneration corresponding to decrease in the T2 weighted sagittal and the appearance of a dark transverse band. This data is quite similar to that observed in humans during the course of IVD degeneration [27]. The decrease in T2 weighted sagittal intensity in human IVD after 20 years of age is well acknowledged and reflects the decrease in water and proteoglycan contents during degeneration process [28]. Histological studies have thus suggested that this dark transverse band may likely be due to the accumulation of fibrous tissue in the central zone of NP [29]. It has been reported that the concordance of the classification system with the morphologic and MRI changes is likely to be high [30]. A high signal intensity of T2 weighted images in MRI is often used indirectly to evaluate water content in the IVD [30]. Modic et al. reported that the signal loss of the disc on T2 weighted MRI scans correlated with the progressive degenerative changes in the IVD [31]. We observed that signal loss in the damaged disc is recovered in the MSC-hydrogel treated group. To further address whether the disc degeneration repair observed by MRI may correlate with some tissue alteration, we then performed histological staining as a method of validation. Histologically, the disc of the MSC-transplanted models had preserved the disc structure with minimal degeneration observed as compared to the untreated damaged discs. We found that the primary morphological features of disc degeneration, cell depletion in the nucleus pulposus, and disorientation of oval annular structure were not present suggesting that the injection of the MSC-hydrogel appears to halt the progression of the disease state. Extracellular matrix, although not proven here, is suspected to originate from the implanted MSCs, which was observed within the interlineating layers. Safranin O staining of MSC-Hydrogel treated group showed high intensity, suggesting the potential for rich matrix production, including proteoglycan from the transplanted cells. This finding appears to be supported by our image analyses, which demonstrate that the increase in collagen type II and aggrecan staining intensity levels was evidently higher in the MSC transplanted models. This strongly suggests that MSCs delay the degenerative process whilst maintaining the proteoglycan content.

Despite the effectiveness of the MSCs in treating the damaged disc, it is worthwhile noting that there are some limitations of this study which have been identified but were unfortunately unavoidable. The smaller sample size may not be convincingly representative and therefore the study should be planned with more number of animals. This would be helpful in predicting more significant statistical changes for the observations made. Nevertheless, the results of this study were meant as preliminary findings and thus serve its purpose of proving the concept that the use of this cell-matrix material may be useful for treating damaged IVD. Another limitation of this study was the fact that the nucleus pulposus was only damaged and not completely removed. Hence, there might be the possibility of native nucleus pulposus to proliferate in the scaffold support and contribute to the treatment effectiveness along with the MSCs. To prove the effectiveness of MSCs, cell tracking through labeled MSCs would be required. Future studies should be conducted to demonstrate these changes to support our current findings.

## 5. Conclusion

The present study has shown that adding MSCs to hyaluronan hydrogel significantly increased the quality of repaired tissue which was not provided by the scaffold alone, which by itself is a merit on its own and must be considered as a good indicator of the success of this proposed therapy. Mesenchymal stromal cells (MSCs) embedded in hyaluronan hydrogel (HyStem) reduce the rate of nucleus pulposus degeneration and to a certain extent result in the reversal of the IVD degeneration process. Although the study is still preliminary, the results warrant further studies to be conducted since the outcome produced may have profound impact to future repair strategies involving damaged IVD.

## Abbreviations

BM-MSCs: Bone marrow derived mesenchymal stromal cells
Col-II: Collagen type II
Acan: Aggrecan
H and E: Hematoxylin and eosin
L-DMEM: Low-glucose Dulbecco's modified eagle medium
mg: Milligram
min: Minute
mL: Milliliter
mm: Millimeter
NP: Nucleus pulposus
MSCs: Mesenchymal stromal cells
nm: Nanometer
Saf O: Safranin O.
